# Real World Outcomes in Patients with Advanced Melanoma Treated in Alberta, Canada: A Time-Era Based Analysis

**DOI:** 10.3390/curroncol28050338

**Published:** 2021-10-05

**Authors:** Rodrigo Rigo, Jordan Doherty, Kim Koczka, Shiying Kong, Philip Q. Ding, Tina Cheng, Winson Y. Cheung, Jose G. Monzon

**Affiliations:** 1 Tom Baker Cancer Centre, Division of Medical Oncology, Cumming School of Medicine, University of Calgary, Calgary, AB T2N 1N4, Canada; Rodrigo.Rigo@albertahealthservices.ca (R.R.); Kim.Koczka@albertahealthservices.ca (K.K.); tina.cheng@albertahealthservices.ca (T.C.); Winson.Cheung@albertahealthservices.ca (W.Y.C.); 2 Gastrointestinal Cancer and Melanoma Clinical Research Program, Cumming School of Medicine, University of Calgary and Alberta Health Services, Calgary, AB T2N 1N4, Canada; 3 Cambridge Memorial Hospital, Cambridge, ON N1R 3G2, Canada; jordan@jdmed.net; 4 Centre for Health Informatics, University of Calgary, Calgary, AB T2N 1N4, Canada; Shiying.Kong@albertahealthservices.ca; 5 Oncology Outcomes Research Initiative, University of Calgary, Calgary, AB T2N 1N4, Canada; p.ding@ualberta.ca; 6 Cancer Control Alberta, Alberta Health Services, Calgary, AB T2N 1N4, Canada; 7 Clinical Research Unit, Tom Baker Cancer Centre, Calgary, AB T2N 1N4, Canada

**Keywords:** melanoma, overall survival, ipilimumab, nivolumab, targeted therapy, *BRAF* mutation, real-world study

## Abstract

Immune checkpoint and MAP kinase pathway inhibitors can significantly improve long-term survival for patients with melanoma. There is limited real-world data of these regimens’ effectiveness. We retrospectively analyzed 402 patients with unresectable and metastatic melanoma between August 2013 and July 2020 treated with immune checkpoint inhibitors and MAP kinase pathway targeted therapy in Alberta, Canada. Overall survival (OS) was compared using Kaplan–Meier and Cox regression analyses. Subgroup survival outcomes were analyzed by first-line treatment regime and *BRAF* mutation status. Three treatment eras were defined based on drug access: prior to August 2013, August 2013 to November 2016, and November 2016 to July 2020. Across each era, there were improvements in median OS: 11.7 months, 15.9 months, and 33.6 months, respectively. Patients with BRAF mutant melanoma had improved median OS when they were treated with immunotherapy in the first line as opposed to targeted therapy (median OS not reached for immunotherapy versus 17.4 months with targeted treatment). Patients with *BRAF* wild-type melanomas had improved survival with ipilimumab and nivolumab versus those treated with a single-agent PD-1 inhibitor (median OS not reached and 21.2 months). Our real-world analysis confirms significant survival improvements with each subsequent introduction of novel therapies for advanced melanoma.

## 1. Introduction

The treatment landscape for melanoma has changed dramatically over the last decade, with major advances in both immune checkpoint inhibitors (ICIs) and targeted treatments. First, two classes of ICIs have demonstrated improvements in overall survival (OS): cytotoxic T lymphocyte antigen (CTLA-4) and programmed cell death protein (PD-1) inhibitors. They work as single agents and in combination by enhancing the ability of T-cells to mount an anti-cancer immune response [1,2,3]. Second, BRAF and MEK inhibitors have also demonstrated improved survival as single agents and in combination [4,5,6,7,8]. They have been designed to target the constitutive activation of the mitogen activated protein kinase (MAPK) pathway, inappropriately activated by the *BRAF* V600 mutation, occurring in around 40% of melanomas [4].

Significant advancements in treatment of advanced melanoma started in 2010, when Ipilimumab, a CTLA-4 inhibitor, demonstrated an improvement in survival [1]. It showed durable responses could be achieved with approximately 20% of patients alive at 10 years [9]. By 2015, a new class of ICIs improved survival in advanced melanoma. The PD-1 inhibitors, pembrolizumab and nivolumab, exhibited superior survival to both ipilimumab and dacarbazine [3,10]. Again, the responses to these single agent PD-1 inhibitors were durable with three-year survival ranging from 40–51% [11,12]. Finally, in 2017, the combination of ipilimumab and nivolumab compared to ipilimumab or nivolumab monotherapy showed superior survival [13]. Updated analysis of this trial showed the five-year survival outcomes of ipilimumab and nivolumab combination to be 52% [14]. These immunotherapies have revolutionized the treatment of advanced melanoma and represent the current standard of care for patients.

During the same period as the development of the ICIs, targeted agents were also examined in patients with advanced melanoma harboring *BRAF* V600 mutations. As single agents the BRAF inhibitors, vemurafenib in 2011 and Dabrafenib in 2012, demonstrated improved survival compared to chemotherapy [4,15]. By 2014, combination treatment with BRAF and MEK inhibitors, either Dabrafenib and Trametinib or Vemurafenib and Cobimetinib, had shown superiority over BRAF inhibition alone [14,16]. Long-term data from the dabrafenib and trametinib trials demonstrated that the five-year overall survival is 34% [7]. Although targeted treatments were not thought to result in durable responses such as ICIs, some subsets of patients treated with dabrafenib and trametinib can have responses lasting years, especially those with low tumor burden, normal LDH at baseline, and good performance status [16].

The advances in melanoma treatment allows patients to receive single agent PD-1 inhibitors or combination Ipilimumab and nivolumab. *BRAF* mutant melanoma patients also have the option of combination BRAF and MEK inhibitors. Considering the results of these trials, we aimed to corroborate the survival benefits seen with these novel treatments in a real world setting by retrospectively examining melanoma patients treated in the Canadian province of Alberta from 2007 to 2020. Given there has never been a published inter-class comparison between these therapies, we also compared outcomes of immunotherapy and targeted BRAF/MEK inhibitors and investigated the difference in survival based on which treatment was received first-line.

## 2. Materials and Methods

### 2.1. Patients

Four hundred and two adult patients with advanced melanoma diagnosed with unresectable Stage III and Stage IV melanoma from 2007 to 2020 were identified from the Alberta Cancer Registry. Demographics were obtained from electronic medical records, including Eastern Cooperative Oncology Group (ECOG) performance status, *BRAF* mutation status, treatments received and survival outcomes. Ethics approval was obtained from the Alberta Cancer Research Ethics Board prior to data collection for this study.

### 2.2. Statistical and Survival Analyses

Outcomes based on era of treatment: We divided the population based on treatment era, defined as when certain drugs became available in Alberta, Canada. Prior to August 2013 the only treatments available for advanced melanoma patients were dacarbazine, ipilimumab, and single agent vemurafenib. Combination dabrafenib and trametinib became available in August 2013, pembrolizumab became available in May of 2014, nivolumab became available in July 2015, and combination ipilimumab and nivolumab became available in November of 2016. All these dates are based on compassionate access programs and clinical trial participation. These programs and clinical trials continued until the drugs were approved for provincial funding and administration.

Based on dates of drug availability, we defined three eras for the total population: prior to August 2013, August 2013–November 2016, and after November 2016. We assessed overall survival outcomes based on these eras and compared it to a cohort who never received treatment.

Similarly, to determine the magnitude of benefit seen over time in the *BRAF* mutation positive and negative cohorts, we also examined distinct eras based on when treatments became available (Table 1).

For the *BRAF* mutant cohort we compared the overall survival of patients treated prior to the availability of dabrafenib and trametinib (prior to August 2013) to after dabrafenib and trametinib (after August 2013) and to those who never received treatment. A similar analysis was performed for advanced *BRAF* wild-type and unknown *BRAF* status melanoma patients. We defined these eras as prior to access to PD-1 inhibitors (prior to May 2014), access to single agent PD-1 inhibitors (May 2014–November 2016), and access to the combination ipilimumab and nivolumab (after November 2016). This was compared to *BRAF* wild-type melanoma patients who never received treatment.

Outcomes based on first treatment received: We analyzed if there was any difference in survival outcomes dependent on first line treatment received by patients that were *BRAF* mutant and *BRAF* wild type. For *BRAF* mutation patients, we only included patients who received treatment after August 2013 when dabrafenib and trametinib were accessible. As for patients with *BRAF* wild-type or unknown melanomas, we compared those patients that received single agent PD-1 inhibitor versus combination ipilimumab and nivolumab.

OS was estimated by using the Kaplan–Meier (KM) method with 95% CI (confidence interval). Analyses were conducted using SAS 9.4 (SAS Institute Inc., Cary, NC, USA). Statistical significance was defined as *p* ≤ 0.05 level, and all tests were two-sided.

## 3. Results

### 3.1. Patient Characteristics

For this retrospective cohort study, we identified a total of 402 patients with unresectable Stage III or Stage IV melanoma treated in the province of Alberta, Canada. The mean age for all patients was 63 years and the majority were male (69.4% vs. 30.6% female). Most patients had an ECOG performance status of 0–1 (63.4%) and 36.6% had a score of ≥2. Most patients had been diagnosed with Stage IV disease (80.1%) and 80 patients (19.9%) with unresectable Stage III disease. *BRAF* mutations were found in 100 patients (24.9%), 137 (34.1%) had unknown *BRAF* status, and 165 (41%) were *BRAF* wild type. Previously described poor prognostic factors such as ECOG ≥ 2, elevated LDH at baseline and Stage 4 disease, are described in Table 2.

### 3.2. Outcomes Based on era of Treatment

**Figure 1:**
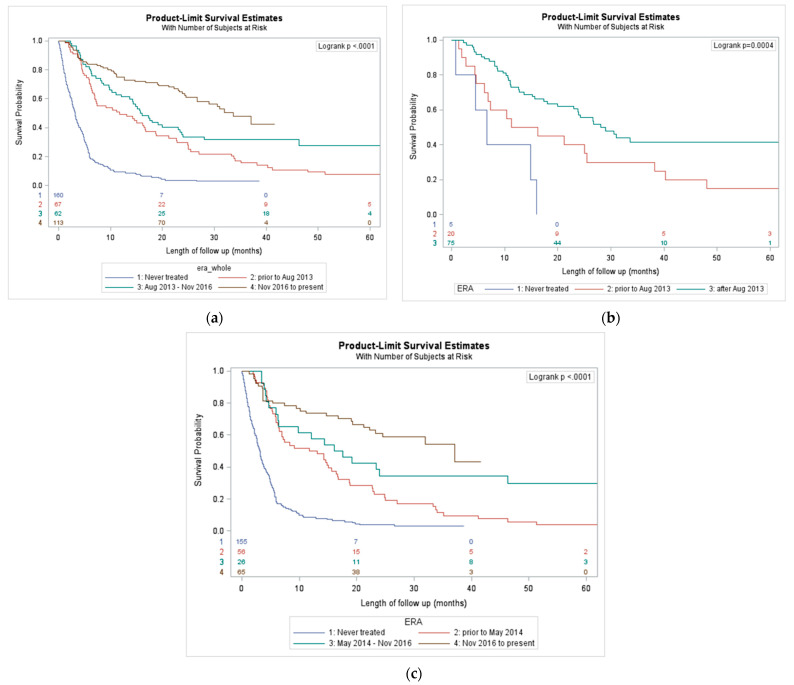
(**a**) Overall survival outcomes by era of novel therapy; (**b**) overall survival outcomes by era in *BRAF* mutant-melanoma; (**c**) overall survival outcomes by era in unknown or wild-type *BRAF* mutation status.

Our analysis revealed improvements in median OS for patients with the successive introduction of novel therapies for all melanoma patients, patients with *BRAF* mutant melanoma, and for patients with *BRAF* wild-type or unknown melanoma. In the total population, from prior to 2013, August 2013–November 2016, and November 2016–July 2020, there were stepwise improvements in median OS, 11.7, 15.9, and 33.6 months respectively (Figure 1a). The median OS of patients that never received treatment was only 3.2 months. Similarly, for *BRAF* mutant melanoma patients, each successive treatment era improved median OS: 13. 7 months prior to August 2013 and 28.1 months after August 2013 (Figure 1b).

*BRAF* mutant melanoma patients that never received treatment had a median OS of only 6.6 months. The same trend appeared for unknown or *BRAF* wild-type patients. Over the successive treatment eras, there was improvement in median OS: 12.4 months for prior to May 2014, 16.8 months for May 2014–November 2016, and 37.0 months for November 2016 to July 2020 (Figure 1c). In *BRAF* wild-type or unknown status patients, those who did not receive any treatment had a median OS was 3.2 months.

### 3.3. Outcomes by First Treatment Received

**Figure 2:**
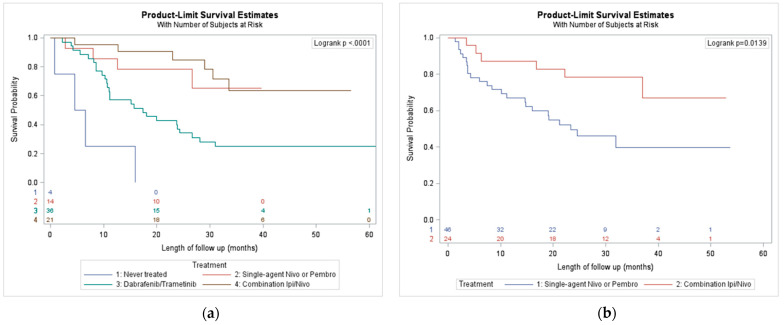
(**a**) Overall survival outcomes by first treatment received of *BRAF* mutation positive; (**b**) overall survival outcomes by first treatment received, in *BRAF* wild type/Unknown.

In the 100 patients with *BRAF* mutant melanoma, the median survival for first line Dabrafenib and Trametinib was 17.4 months (Figure 2a). The median survivals were even better in the first-line immunotherapy groups. The median survival was not reached in the first-line PD-1 inhibitors or combination ipilimumab and nivolumab arms, however, the one-year survival for ipilimumab and nivolumab was superior to the single-agent PD-1 inhibitor groups.

In the patients with *BRAF* wild-type or *BRAF* status unknown, first-line combination ipilimumab and nivolumab was superior to first-line treatment with single agent PD-1 inhibitors (median OS not reached and 23.4 months respectively) (Figure 2b).

## 4. Discussion

Over the last decade, there have been multiple practice changing clinical trials for patients with advanced melanoma. It is important to corroborate these clinical trial results with real-world data as patients are not necessarily reflected in the strict eligibility and exclusion criteria of clinical trials. As a result, the magnitude of benefit observed in a clinical trial may not be observed in a real-world setting. Moreover, because targeted and ICIs were developed independently at the same time, there is no study comparing which treatment is superior, nor evidence identifying the optimal sequence of these treatments. Analyzing real world data may help to answer some of these questions.

This study corroborates the improvement in survival observed when each successive treatment became available in Alberta, Canada. With the introduction of each treatment, there were corresponding incremental improvements in median overall survival. For the total population, the best median OS was 33.6 months, and occurred between November 2016 to July 2020. This era marks the period during which the full current complement of treatments became available. At this time, the combination ipilimumab and nivolumab, dabrafenib and trametinib, and the single agent PD-1 inhibitors pembrolizumab and nivolumab were all available and funded.

In the registration trials for these treatment options, the best survival is reported in the CHECKmate-067 study, which showed a 5-year OS of 52% with first line ipilimumab and nivolumab treatment in advanced melanoma patients [14]. The inferior survival reported in this real-world analysis as compared to the Checkmate-067 trial may be due to poor prognostic factors such as ECOG ≥2, patients with active brain metastases, high baseline LDH, previous treatment, and a higher proportion of patients with Stage IV disease (See Table 2). Another real-world analysis of melanoma patients noted similar incremental improvements in survival with patients diagnosed with advanced melanoma in 2012, 2014, 2016 [17]. In that study, patients were separated into ‘trial-like’ and ‘trial-excluded’ groups based on the common trial eligibility criteria. In the ‘trial-like’ population, the median OS was not reached in 2016 versus 18.8 months in 2014 and 16.5 months in 2012. In the ‘trial-excluded’ population, the median survivals for the patients diagnosed in 2012, 2014 and 2016 were 4.2, 5.2 and 6.9 months, respectively. In their analysis, 61% of patients were “trial excluded”, which explains the inferior outcomes observed in real-world analyses and underscores the importance of corroborating clinical trial results with the real-world context.

For the *BRAF* wild-type and unknown population, improvements in survival were observed with the introduction of ipilimumab, single agent PD-1 inhibitors, and combination ipilimumab and nivolumab. In the era when combination ipilimumab and nivolumab became available (after November 2016), the median survival was 37.0 months, almost identical to the median survival observed in the *BRAF* wild-type population of the CHECKmate-067 study. For the *BRAF* mutant population, there was an improvement in survival in the era after combined dabrafenib and trametinib became available compared to the era of when only single agent BRAF inhibitors were available (28.1 months and 13.7 months). Interestingly, our *BRAF* mutant cohort OS of 28.1 months is remarkably close to the median survival of 25.9 months in the COMBI-V study which demonstrated a superior OS with dabrafenib and trametinib versus single agent vemurafenib [7]. It is reassuring to corroborate the improvements in overall survival in the *BRAF* mutant along with the *BRAF* wild-type and unknown populations when they are independently analyzed.

There is currently no clinical trial data to determine what is the optimal sequence for treating advanced *BRAF* mutant melanoma patients. In this real-world analysis, our data suggest that upfront immunotherapy with single agent PD-1 inhibitors or combination ipilimumab and nivolumab had better survival outcomes as compared to upfront dabrafenib and trametinib. One potential confounder for patients treated with immunotherapy having better outcomes is that single agent PD-1 inhibitors and combination immunotherapy did not become available until May 2014 and after November 2016, while combination dabrafenib and trametinib became available in 2013. It is possible, that the patients with *BRAF* mutant melanoma, treated with targeted therapy had worse outcomes because there was no access to PD-1 inhibitors or combination immunotherapy after progression, however, pembrolizumab was available within nine months of dabrafenib and trametinib access. Another possibility is that due to the rapid responses with targeted treatment, patients with poorer prognostic factors (such as rapidly progressing disease, heavy burden of disease, elevated LDH, etc.) were preferentially started on targeted treatment. Lastly, upfront immunotherapy may indeed be superior to targeted agents in the first line setting and another real-world analysis has demonstrated similar trends in this scenario [18].

Our real world-data shows some discrepancies compared the CHECKmate-067 study, where the 3-year survival with ipilimumab and nivolumab was highest in the *BRAF* mutant patients (60%) as compared to the *BRAF* wild-type patients (48%). The three-year survival with single agent nivolumab in the *BRAF* mutant group (46%) only modestly outperformed the wild-type group (43%). In our real-world analysis, first-line ipilimumab and nivolumab combined had a three-year survival of 71% and 79%, for the *BRAF* mutant and wild-type groups, respectively. Similarly, single agent PD-1 inhibitor in the first-line setting had a three-year survival of 71% compared to 48% for the *BRAF* mutant and wild-type groups. Possible reasons for these discrepancies are the retrospective nature of this analysis and the small sample size of each of the cohorts. These findings highlight that further research into predictive factors for combination immunotherapy versus single agent PD-1 inhibitor is required. This is especially important given the increased toxicity observed with ipilimumab and nivolumab versus single agent PD-1 inhibitor treatment. The SECOMBIT trial (NCT02631447) is evaluating the optimal sequencing of treatment in advanced *BRAF* mutant melanoma patients by randomizing patients 1:1:1 to first-line targeted BRAF and MEK inhibitors followed by ipilimumab and nivolumab at progression; upfront ipilimumab and nivolumab followed by targeted treatment at progression; or an initial run-in period of targeted treatment for eight weeks, followed by ipilimumab and nivolumab until progressive disease, then a switch back to targeted treatment [19,20]. Hopefully this trial will definitively answer what the optimal treatment strategy is, and if an initial run-in period of targeted treatment can enhance the effects of combined ICIs.

Finally, our real-world analysis confirmed significant survival improvements with the introduction of novel therapies for advanced and metastatic melanoma over the last decade. The best treatment era over the last decade corresponds to when combined ipilimumab and nivolumab became available. This era also represents the current standard of care options in the province of Alberta, in which patients can access ipilimumab and nivolumab combination or single agent PD-1 inhibitor and targeted treatment with dabrafenib and trametinib. This real-world analysis also demonstrated that first-line immunotherapy produced better survival outcomes compared to upfront targeted treatment in patients with *BRAF* mutation positive melanoma. Lastly, there was a larger magnitude of benefit with combination immunotherapy as compared to single agent PD-1 inhibitor in the *BRAF* wild-type as opposed to the *BRAF* mutant groups, highlighting the need for more predictive tools to guide our use of combination versus single agent ICIs. Although this data suggest that first-line combination immunotherapy is the treatment of choice in the BRAF wildtype and mutant populations, a personalized approach is always warranted taking into account a variety of factors, such as the patient’s age, burden and pace of disease, comorbidities, and performance status.

## Figures and Tables

**Table 1:** Treatment Eras and New Agent Demographics.

Variables	Category	Total	1st Era (Mar 2007–July 2013)	2nd Era (August 2013–November 2016)	3rd Era (November 2016–July 2020)	Never Treated	*p*-Value
Number of patients		402	67	62	113	160	
Treatment			DTIC Ipilimumab Vemurafenib	Pembrolizumab Nivolumab Dabrafenib/ Trametinib	Ipilimumab/ Nivolumab		
Age	Mean (STD)	63 (15)	68 (15)	55 (14)	59 (14)	65 (13)	<0.0001
Sex	Female	123 (30.6%)	52 (32.5%)	19 (28.4%)	17 (27.4%)	35 (31.0%)	<0.0001
	Male	279 (69.4%)	108 (67.5%)	48 (71.6%)	45 (72.6%)	78 (69.0%)	
*BRAF* mutation status	Mutant	100 (24.9%)	5 (3.1%)	20 (29.9%)	27 (43.5%)	48 (42.5%)	<0.0001
	Wild	165 (41.0%)	53 (33.1%)	29 (43.3%)	35 (56.5%)	48 (42.5%)	
	Unknown	137 (34.1%)	102 (63.7%)	18 (26.9%)	0 (0.0%)	17 (15.0%)	

**Table 2:** Patient Demographics.

Variables	Category	Total (*n* = 402)	Alive (*n* = 94)	Death (*n* = 308)	*p*-Value
Age	Mean (STD)	63 (15.3)	63.1 (13.4)	63 (15.9)	
Sex	Female	123 (30.6%)	33 (35.1%)	90 (29.2%)	
	Male	279 (69.4%)	61 (64.9%)	218 (70.8%)	
ECOG	0–1	255 (63.4%)	86 (91.5%)	169 (54.9%)	<0.0001
	2+	147 (36.6%)	8 (8.5%)	139 (45.1%)	
Melanoma stage	Stage III	80 (19.9%)	44 (46.8%)	36 (11.7%)	<0.0001
	Stage IV	322 (80.1%)	50 (53.2%)	272 (88.3%)	
*BRAF* mutation	Mutant	100 (24.9%)	39 (41.5%)	61 (19.8%)	<0.0001
	Wild	165 (41%)	41 (43.6%)	124 (40.3%)	
	Unknown	137 (34.1%)	14 (14.9%)	123 (39.9%)	
LDH	LDH high	140 (34.8%)	6 (6.4%)	134 (43.5%)	<0.0001
	LDH not high	166 (41.3%)	66 (70.2%)	100 (32.5%)	
	Unknown	96 (23.9%)	22 (23.4%)	74 (24%)	

## Data Availability

The data presented in this study are available on request from the corresponding author.
